# The iMPROVE Study; Design, Dietary Patterns, and Development of a Lifestyle Index in Overweight and Obese Greek Adults

**DOI:** 10.3390/nu13103495

**Published:** 2021-10-03

**Authors:** Maria Kafyra, Ioanna P. Kalafati, Efthymia A. Katsareli, Sophia Lambrinou, Iraklis Varlamis, Andriana C. Kaliora, George V. Dedoussis

**Affiliations:** 1 Department of Nutrition and Dietetics, School of Health Sciences and Education, Harokopio University of Athens, 17671 Athens, Greece; mariakaf@hua.gr (M.K.); nkalafati@gmail.com (I.P.K.); ekatsa@hotmail.com (E.A.K.); sophialambrinou@gmail.com (S.L.); akaliora@hua.gr (A.C.K.); 2 Department of Informatics and Telematics, School of Digital Technology, Harokopio University of Athens, 17671 Athens, Greece; varlamis@hua.gr

**Keywords:** overweight, obesity, adults, dietary patterns, lifestyle index, health status, online assessment tool, nutritional intervention, weight management

## Abstract

Background: Dietary and lifestyle habits constitute a significant contributing factor in the formation of anthropometric and biochemical characteristics of overweight and obese populations. The iMPROVE study recruited overweight and obese Greek adults and investigated the effect of gene–diet interactions on weight management when adhering to a six-month, randomized nutritional trial including two hypocaloric diets of different macronutrient content. The present paper displays the design of the intervention and the baseline findings of the participants’ dietary habits and their baseline anthropometric and biochemical characteristics. Methods: Baseline available data for 202 participants were analyzed and patterns were extracted via principal component analysis (PCA) on 69-item Food-Frequency Questionnaires (FFQ). Relationships with indices at baseline were investigated by multivariate linear regressions. A Lifestyle Index of five variables was further constructed. Results: PCA provided 5 dietary patterns. The “Mixed” pattern displayed positive associations with logBMI and logVisceral fat, whereas the “Traditional, vegetarian-alike” pattern was nominally, negatively associated with body and visceral fat, but positively associated with HDL levels. The Lifestyle Index displayed protective effects in the formation of logBMI and logGlucose levels. Conclusions: Dietary patterns and a Lifestyle Index in overweight and obese, Greek adults highlighted associations between diet, lifestyle, and anthropometric and biochemical indices.

## 1. Introduction

The past decades have marked a noticeable increase in adult overweight and obesity. Current epidemiological evidence suggests that more than half of the adult in the European population presents a body mass index (BMI) of above 25 kg/m^2^ and is, therefore, classified in the category of overweight [1]. Factors relating to increased body weight vary, including genetic predisposition, lifestyle habits, and environmental conditions, as well as their respective interplay.

The role of dietary habits in overweight and obesity development has been extensively studied in populations of various ages and ethnicities [2]. It is widely established that consumption of energy-dense foodstuffs and/or products with high sugar or fat content is positively correlated with increased weight and weight management [3]. Apart from habitual dietary preferences, adherence to specific dietary patterns, such as the Mediterranean or the Western diets, has also been associated with long-term weight management. In their 2017 review, Mu et al. demonstrated that diets rich in fruits and vegetables are correlated with lower values of BMI, whereas higher consumption of meats and high-fat products are associated with increased BMI [2]. In the same context, Cena and Calder examined the components of constructing a healthy diet and its relation to general health status. Their 2020 review concluded that diets including fruits, vegetables, and plant-based foods, among others, such as the Mediterranean and Asian diets, reduce the risk of non-communicable diseases (NCDs) development [4].

Additionally, the identification of adherence to specific dietary patterns has been shown to be related to lifestyle parameters, namely depression and sleep characteristics. Li et al. showed that adherence to balanced dietary patterns including whole-grain products, fish, fruit, and vegetables was related to decreased risk of depression appearance, in contrast with adherence to western-diet alike patterns with elevated content in processed products and red meat [5]. Moreover, Godos et al. (2021) showed that attrition to similar, balanced dietary patterns was associated with enhanced sleep habits and sleep quality [6].

The effect of dietary habits has recently been incorporated in the attempt to collectively evaluate various lifestyle characteristics, via the construction of Lifestyle Indices (LI). Creation of LI is gaining more and more ground in recent literature and allows for the evaluation of the interconnected effect of multiple variables on phenotypic traits, such as obesity, cognitive abilities [7], development of other NCDs, such as cardiovascular disease [8] and overall mortality rates. Research studies including dietary habits in lifestyle indices are currently gaining more ground. Navarro et al., demonstrated the relationship between a maternal healthy lifestyle index, including calculation of the Healthy Eating Index (HEI), increased physical activity, pre-pregnancy BMI, smoking and alcohol drinking habits and the development of obesity in their offspring. Increased values of the index were negatively associated with obesity development during childhood [9]. Moreover, higher scores of a lifestyle index comprising of age, sex, smoking, drinking and exercise habits, sleep quality and BMI were associated with increased absolute mortality risk of older adults in Europe, United States, and the United Kingdom [10].

In this context, the present analyses represent the baseline results deriving from the iMPROVE study. The iMPROVE study, as a whole, attempts to evaluate gene–diet interactions in observed weight management, weight loss, body composition, and the lifestyle characteristics of a sample of overweight and obese Greek adults, adhering to one out of two hypocaloric dietary regimens of different macronutrient content, for a total duration of 6 months. The present article seeks to: (a) display the design, as well as the aims and objectives of the iMPROVE study and (b) evaluate the sample population’s baseline dietary habits and potential relations with biochemical biomarkers. These analyses constitute the first step in assessing the effect of dietary habits on the participants’ observed weight loss and further associate them with characteristics of genetic predisposition.

## 2. Materials and Methods

The Greek iMPROVE study constitutes a six-month randomized clinical trial, a nutritional intervention focusing on the investigation of gene–diet interactions on body weight regulation and lifestyle parameters. More specifically, the study aims at evaluating the role of target-genes in overweight and obese, Greek adults, under different dietary interventions. The study was approved by the Research Ethics Committee of Harokopio University of Athens (Protocol Number: 1800/13-06-2019) and was conducted at the premises of Harokopio University during the period 2020–2021. Recruitment took place in spring 2020 and handling and analysis of the data was, then, carried out during 2021. Moreover, the study was registered with the ClinicalTrials.gov database of the United States of America National Health Institute’s (NIH) National Library of Medicine (ClinicalTrials.gov Identifier: NCT04699448). Due to the inclusion of human participants throughout its entirety, the trial was conducted adhering to the principles outlined in the Declaration of Helsinki. The study consisted of a sample of 202 overweight and obese, Greek adults, residing at the region of Attica at the time of recruitment. In this context, the present article summarizes the baseline characteristics of the study population and the associations observed between the participants’ dietary habits and biomarkers of glycemic and lipidemic control at baseline.

### 2.1. Nutritional Intervention Design and Study Population

**Figure 1:**
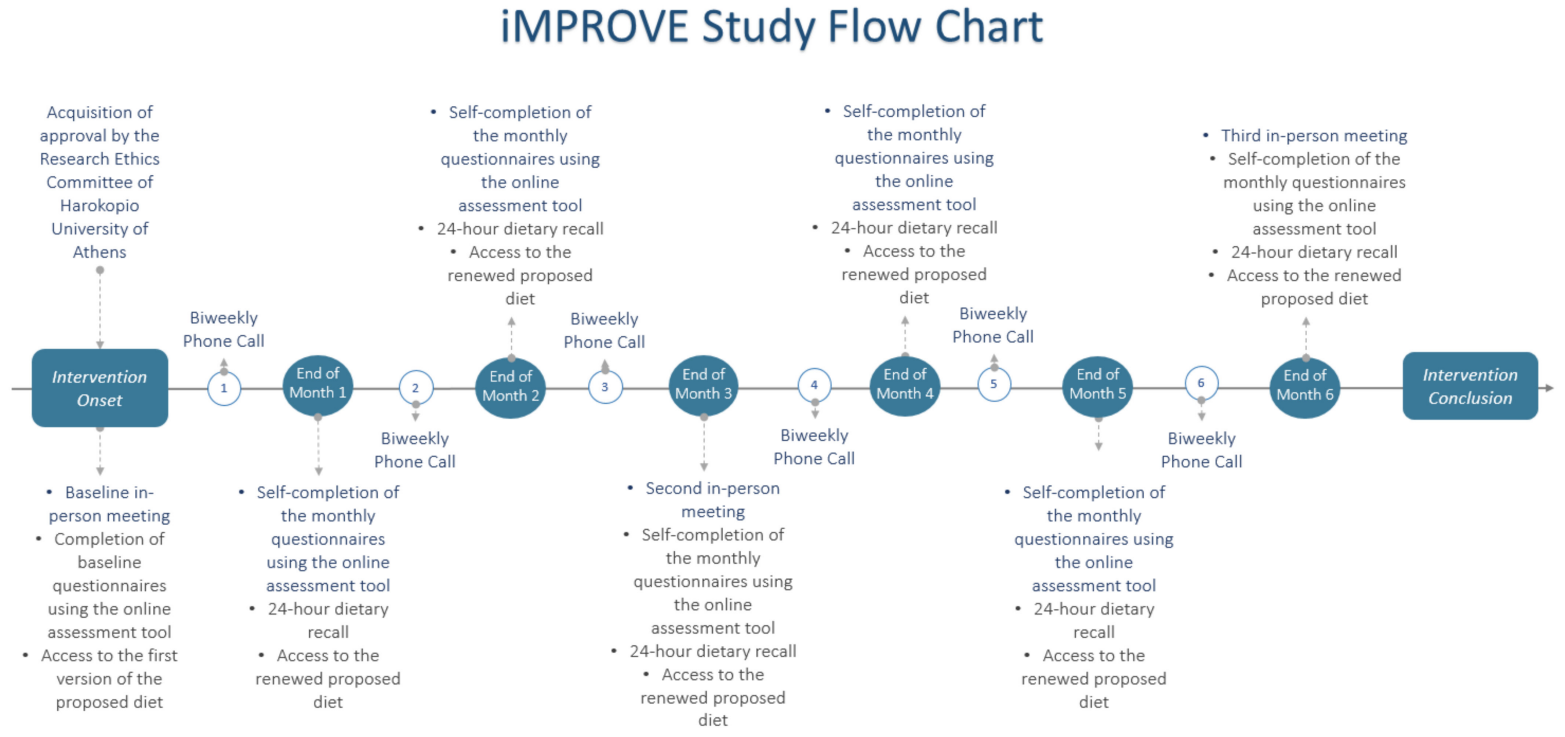
Flow chart of the iMPROVE Study. After completion of the baseline meeting, each participant was randomly allocated to one of the two proposed dietary intervention groups. Those referred either to a dietary regimen high in carbohydrates/low in fat, with 60% of energy intake deriving from carbohydrates, 18% deriving from protein, and 22% deriving from fat, or a dietary regimen high in protein, with 40% of the total energy intake coming from carbohydrates, 30% deriving from protein, and 30% deriving from fat. Nutritional needs for each participant were calculated using the Mifflin equation and adjusted for their reported physical activity level (PAL). According to the guidelines of the National Institutes of Health (NIH) [11], optimal weight loss should take place aiming at a mean weight loss of 0.5 to 1 kg per week, which is translated to a 7500 kcal reduction in the individual’s weekly energy intake, or a 500 kcal reduction on a daily basis. Therefore, daily individual nutritional needs for each participant were further reduced by 500 kcal. The team of Harokopio University of Athens created three categories of proposed hypocaloric diets with a mean energy content of 1500 kcal, 1800 kcal, or 2000 kcal. Six different versions of the proposed diets, one for each intervention month, were created to allow for monthly renewal of the proposed diets, based on the participants’ observed weight loss and dietary habits and/or tastes. The proposed diets concerned a 7-day pattern to be repeated for each month and mainly adhered to the Mediterranean pattern, including the daily consumption of 3 to 5 portions of fruit and vegetables and 1 to 2 portions of dairy products and the weekly consumption of 2 to 3 portions of grains, fish, and chicken, while limiting the consumption of red meat to 1 meal per week.

The overall aims of the iMPROVE study are summarized in the following: (a) To investigate the effect of adhering to two hypocaloric dietary regimens of different macronutrient content on the observed weight loss of overweight and obese adults; (b) to investigate gene–diet interactions concerning the weight management and various lifestyle characteristics of the study population; and (c) to investigate the utility of an original, online assessment tool as a way of long-distance evaluation of the volunteers’ progress. In this spectrum, the objectives of the study were formed as follows: (a) To design a six-month, randomized clinical trial including overweight and obese adults, who were blindly recruited to following one out of two different hypocaloric dietary regimens and who were subject to two in-person follow-up meetings, conducted at three and six months (middle and end) after the beginning of the intervention; and (b) to create an original assessment tool to further monitor the monthly, self-reported progress of the volunteers throughout their participation in the intervention. The design of the study is shown in Figure 1.

Inclusion criteria for participation in the study consisted of: (a) age above 18 years old at the time of recruitment; (b) existence of a body mass index (BMI) of above 25 kg/m^2^; (c) no reporting of extreme weight loss in the 3 to 6 months prior to the beginning of the intervention; and (d) maintenance of a stable level of physical activity prior to and throughout the duration of the intervention. Similarly, the exclusion criteria included: (a) For women, the existence of pregnancy or lactation, or intention of becoming pregnant in the 6 months during the intervention period; (b) the existence of diagnosed comorbidities related to increased body weight or disturbed dietary intake (i.e., diagnosed type 1 or 2 diabetes mellitus, cardiovascular disease, gastrointestinal disorders, mental illness or disorders related to dietary intake); (c) parallel intake of dietary supplements aiming at body weight loss; and (d) parallel participation in a different research study related to weight management and/or dietary intake.

Prospective participants received oral information on the proceedings and the aims of the trial and provided written consent prior to enrolment. All volunteers were asked to visit the premises of Harokopio University of Athens in order to attend the baseline assessment session and the two subsequent, in-person follow-up meetings at the middle and end of the intervention period. All meetings were conducted in the presence of a nutrition health-care professional (dietitian/nutritionist). All in-person sessions included clinical examination, anthropometric measurements, and collection of fasting blood samples. Participants were expected to use the originally created online assessment tool in order to complete questionnaires regarding anthropometric and lifestyle parameters at baseline and at the end of each month. Following successful completion of all monthly questionnaires, the participants were allowed access to the proposed dietary regimen at the beginning of each intervention month.

**Scheme 1:**
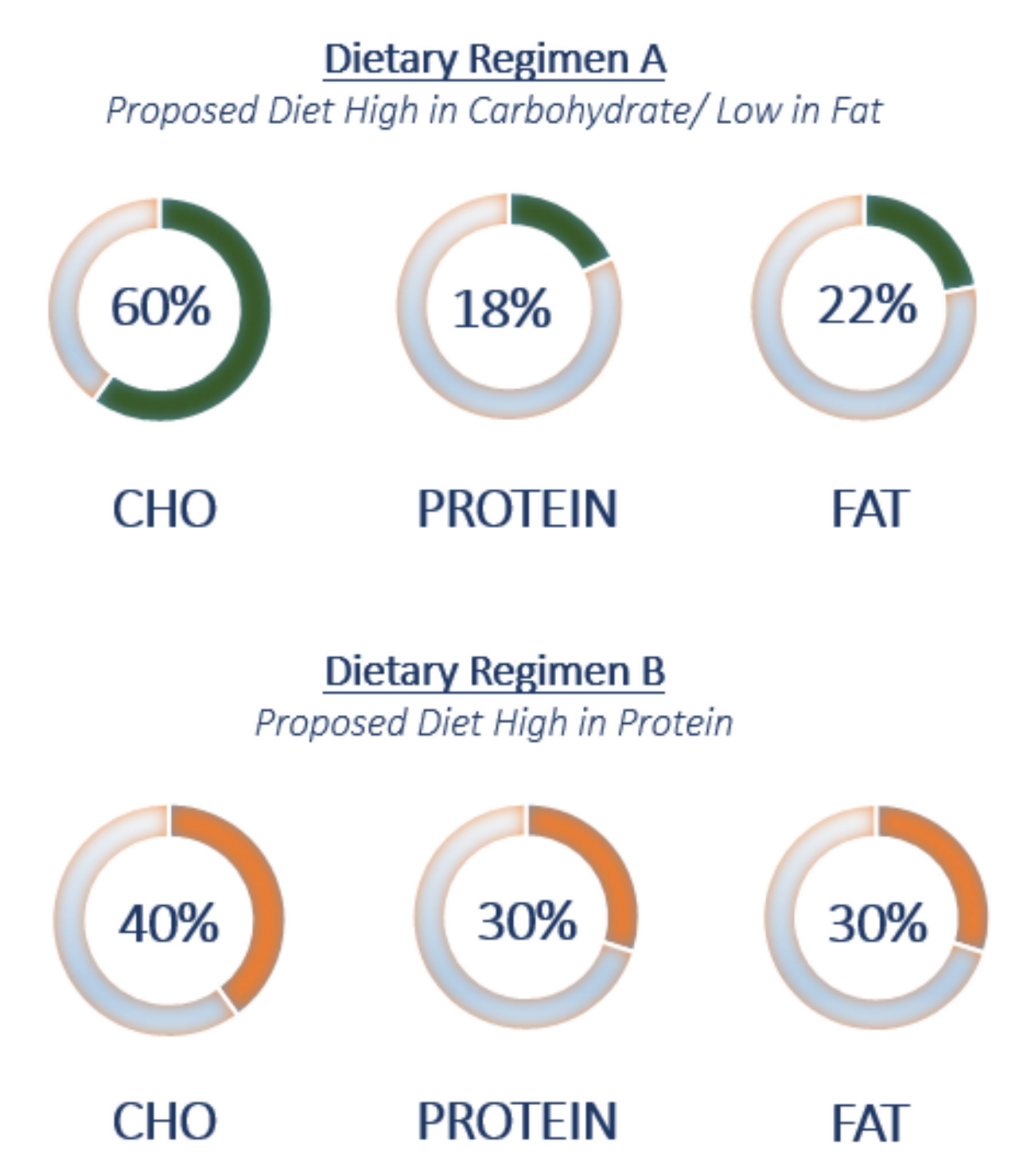
Macronutrient distribution of the proposed diets.

Furthermore, each participant was allocated a nutrition-expert contact who monitored their adherence to the patterns (Scheme 1) and progress by: (a) Conducting biweekly follow-up phone calls and monthly 24-h dietary recalls in order to discuss the potential concerns and provide advice, as to ensure maximum adherence to the proposed diet, (b) monitoring the monthly completion of all online questionnaires; (c) evaluating the participant’s self-reported monthly weight; and (d) renewing and allowing access to the proposed diet at the online platform.

### 2.2. Online Assessment Tool

For the purposes of the study, the team of Harokopio University of Athens created an online assessment tool including all the questionnaires that required completion from the volunteers, both at baseline and at a monthly level. The proposed diet was administered to the participant via access to the platform and was renewed monthly, once all questionnaires of the preceding month were checked by the nutrition expert. Access to the original, online assessment tool was granted with unique usernames and passwords for each volunteer. During the baseline session, each volunteer was taken on a virtual tour of the online assessment tool and was explicitly shown how to access and use it, by the nutrition expert.

At baseline, participants were required to complete questionnaires providing information on: (a) their medical history; (b) demographic characteristics; (c) feeling of satiety, by completing a 5-scale short questionnaire; (d) adherence to the Mediterranean dietary pattern, by completing the questionnaire of the MedDiet Score [12]; (e) depression characteristics, by completing the DEPR-S-10 Questionnaire [13]; (f) characteristics of quality of life and health status, by completing the short version of the SF-12 Questionnaire [14]; (g) characteristics of quality of sleep, by completing the Athens Insomnia Scale Questionnaire [15]; (h) dietary habits, by completing a 69-item Food Frequency Questionnaire (FFQ) [16]; and (i) physical activity habits, by completing the short version of the IPAQ Questionnaire [17]. Completion of the SF-12, sleep and IPAQ Questionnaires at a monthly basis were also a prerequisite prior to the renewal of the proposed diet. Moreover, at the end of each month participants were further called to insert information on their anthropometric measurements (i.e., current weight, waist and hip circumference measurements), feeling of satiety and self-reported adherence to the proposed diet during the past month.

### 2.3. Clinical Examination and Anthropometric Measurements

During all three in-person meetings, clinical examination and anthropometric measurements were carried out by the trained dietitians or health-care or nutrition experts, using suitable equipment and standardized techniques. Clinical examination of the participants included: (a) evaluation of their physical status; and (b) blood pressure measurements, conducted after ensuring that the participant was at a calm state and at their bare, left upper arm, while sitting in an upright position with elevated feet and the arm supported at heart level.

The anthropometric data collected included: (a) Height measurements to the nearest 0.1 cm, using a portable stadiometer, where the participant was barefoot, with relaxed shoulders and looking straight ahead; (b) weight measurements to the nearest 0.1 kg, using the scales of the Tanita BC-418 Segmental Body Composition Analyzer, where the participant was barefoot and maintaining light clothing; (c) waist measurements (between the twelfth rib and the iliac crest), using a non-extensible soft tape; and (d) hip measurements, at the widest point of the hips, using a non-extensible soft tape. Participants were further shown and taught how to properly conduct the waist and hip circumference measurements, in order to monitor their progress and report it in the monthly anthropometric measurements’ online questionnaire. Body composition analysis for individual participants was conducted via bioelectrical impedance analysis and more specifically, by using the Tanita BC-418 Segmental Body Composition Analyzer. The participants were refrained from food or water intake for at least two hours prior to the measurement conduct, be barefoot and maintain light clothing without any metal objects. Body composition data were acquired for each volunteer including body fat percentage, amount of body fat in kilos, distribution of body fat in the trunk and the limbs, body water percentage, and fat free mass. Body mass index (BMI) was calculated via dividing the weight (kg) by the square height (in m^2^) for each participant and subjects were classified to the categories of: overweight (25 kg/m^2^ ≤ BMI < 30 kg/m^2^), obese with class I obesity (30 kg/m^2^ ≤ BMI < 35 kg/m^2^), obese with class II obesity (35 kg/m^2^ ≤ BMI < 40 kg/m^2^), or obese with class III obesity (BMI ≥ 40 kg/m^2^).

### 2.4. Biochemical Analyses

Upon arrival to an in-person meeting and after following an 8-h fasting, a blood sample of 23 mL was collected from the antecubital vein of the participant by a trained health-care professional, following blood pressure measurements. Hematological biomarkers and biomarkers of biochemical profile were analyzed. All remaining samples were stored at −80 °C for future analyses. Low-density cholesterol (LDL-C) was calculated using the Friedewald Equation.

### 2.5. Genotyping Analyses

The buffy coat samples isolated for each participant were used for DNA extraction, via use of the Invitrogen iPrep Purification Instrument and the Invitrogen iPrep PureLink gDNA Blood Kit [18]. Isolated samples were stored at −20 °C for a period of up to two months after extraction and prior to genotyping. Samples were stored at −80 °C, for a longer period and future analyses. All samples were sent for genome-wide sequencing using the Axiom Precision Medicine Diversity Research Array [19], which provided data for over 850,000 SNPs, deletions, and CNVs.

### 2.6. Dietary Assessment

Assessment of dietary intake at baseline took place by completing the validated 69-item Food Frequency Questionnaire (FFQ) in the online assessment tool. Dietary assessment and evaluation of subsequent adherence to the proposed diet was conducted monthly via: (a) A 24-h dietary recall, carried out by the nutrition expert; and (b) completion of the 5-scale self-reported adherence questionnaire, via use of the online assessment platform.

### 2.7. Physical Activity Assessment

Physical activity levels at baseline were assessed by completing the short version of the International Physical Activity Questionnaire (IPAQ) on the online platform. The same questionnaire was completed at the end of each intervention month.

### 2.8. Statistical Analysis

Data analyses were conducted using the Statistical Package for Social Sciences (SPSS), version 23 [20], as well as the R statistical package [21]. Dietary patterns were extracted by conducting principal component analysis (PCA) on 32 food groups deriving from the data of the FFQ. The Varimax orthogonal rotation was used and the KMO and Bartlett’s test was implemented to evaluate data adequacy. Five dietary patterns were set to be extracted with Eigen values bigger than 1. Variable distribution was evaluated via the Shapiro–Wilk and Kolmogorov–Smirnov tests. Differences in mean/median values of variables within the two sexes were evaluated using the Mann–Whitney test. We further tested for potential associations between the extracted patterns and a variety of anthropometric and biochemical indices, via multivariate linear regressions. Non-normally distributed variables were log-transformed. We further examined the potential associations between the extracted patterns and several indices, by separating them into tertiles and testing for associations using the parametric ANOVA test and the non-parametric Kruskal–Wallis test, depending on the distribution of the examined variable. After analyzing the data for the entirety of the sample, a novel Lifestyle Index was constructed including variables found to be correlated with logBMI and body fat percentage, based on Pearson’s chi-square test values. Further association tests (i.e., multivariate regressions) were conducted to assess the potential relationship between the Lifestyle Index and clinical and biochemical biomarkers. The level of statistical significance for all analyses was set at α *=* 0.05 and results were also interpreted for the adjusted cut-off value of a *=* 0.05/number of patterns extracted (i.e., a *=* 0.05/5 *=* 0.01).

## 3. Results

### 3.1. Descriptive Statistics

The entirety of the study population’s anthropometric, clinical, dietary, and lifestyle characteristics are presented in Table 1 and Table 2. Median ± IQR is presented for all non-normally distributed variables and mean ± standard deviation (SD) is presented for the variables following the normal distribution. Out of the 235 volunteers expressing interest to participate in the study, data are shown for 202 eligible subjects who successfully attended the baseline meeting, completed the majority of the baseline questionnaires using the online tool, and were recruited in one of the two intervention arms. The sample size of the 202 individuals assures adequate power to detect statistical significance. Our baseline sample consisted of 142 women (70.29%) and 60 men (29.7%), with a median age of 47 years old. The majority of participants were married (60.9%), with more than half of our sample reporting having higher education (61.9%) and less than 3% reporting having no acquired education at all. The vast majority of the participants were reported as non-smokers (151 non-smokers vs. 50 smokers, out of 201 participants with available data). The estimated physical activity level showed that roughly half of the subjects were leading a moderately active way of life (104 out of the 199 participants with available data), with about 32% reporting a sedentary lifestyle. All 202 eligible volunteers were blindly recruited in the intervention groups, with 46.5% following the high-carbohydrate/low-fat diet and 53.5% adhering to the high-protein diet.

Overweight participants constituted 38.6% of our overall sample, with the remaining 61.4% spreading across the three different obesity categories (35.1%, 14.9%, and 11.4% of the participants classified as Class I, II, or III obese, respectively). Median BMI was calculated at 31.34 kg/m^2^ and did not differ between men and women, whereas body composition data displayed statistically significant differences between the two sexes, with men reporting higher levels of fat-free mass (71.20 kg vs. 48.50 kg, *p* < 0.001) and total body water (*p* < 0.001) and women presenting increased body fat values (41.40% vs. 29.55%, *p* < 0.001). Although men were found to have increased waist-to-hip ratio (WHR) (0.92 vs. 0.83, *p* < 0.001) waist circumference in comparison to women (105 cm vs. 96.5 cm, *p* < 0.001), hip circumference did not present significant differences. Similar differences were further observed in the clinical characteristics, with men reporting higher levels of blood pressure, fasting glucose (95 mg/dL vs. 92 mg/dL, *p* < 0.001), serum urea (30.12 mg/dL vs. 27.00 mg/dL, *p* < 0.001), creatinine (0.12 mg/dL vs. 0.10 mg/dL, *p* < 0.001), uric acid levels (5,75 mg/dL vs. 4.30 mg/dL, *p* < 0.001), total triglycerides (105.5 mg/dL vs. 86 mg/dL, *p* < 0.001), serum bilirubin (0.45 mg/dL vs. 0.35 mg/dL, *p* < 0.001), SGOT (18 IU/L vs. 15 IU/L, *p* < 0.001), and SGPT levels (22.00 IU/L vs. 13 IU/L, *p* < 0.001). Women presented higher levels of HDL cholesterol (52.00 mg/dL vs. 42.00 mg/dL, *p* < 0.001) and serum albumin levels (4.40 g/dL vs. 4.20 g/dL, *p* < 0.001).

### 3.2. Lifestyle Characteristics

The 8-item Athens Insomnia Scale (AIS) score on evaluation of sleep qualities was calculated for participants who reported the selected outcomes more than three times per week in the month leading to the beginning of the intervention. The AIS score presented a median of 5 out of the scale maximum scoring of 24 and did not display statistically significant differences between the sexes. Overall, participants did not express significant irregularities neither in sleep quality, including sleep induction, total sleep duration, and awakenings at night and expressed delayed sleep induction, nor in effects of sleep on aspects of the next day (i.e., well-being, overall functioning, and sleepiness).

**Figure 2:**
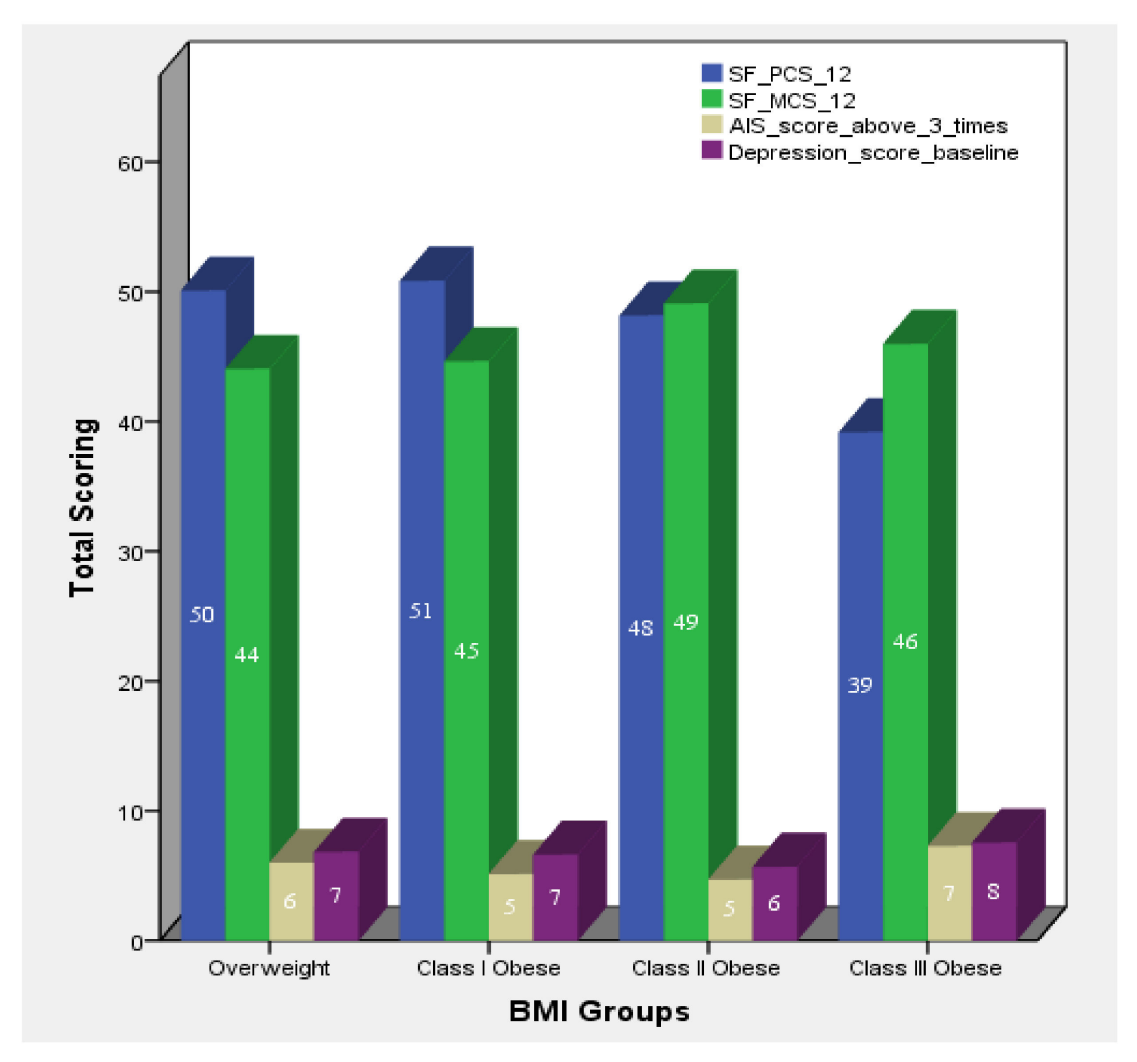
Baseline scoring of the four lifestyle questionnaires, based on BMI categories.

**Figure 3:**
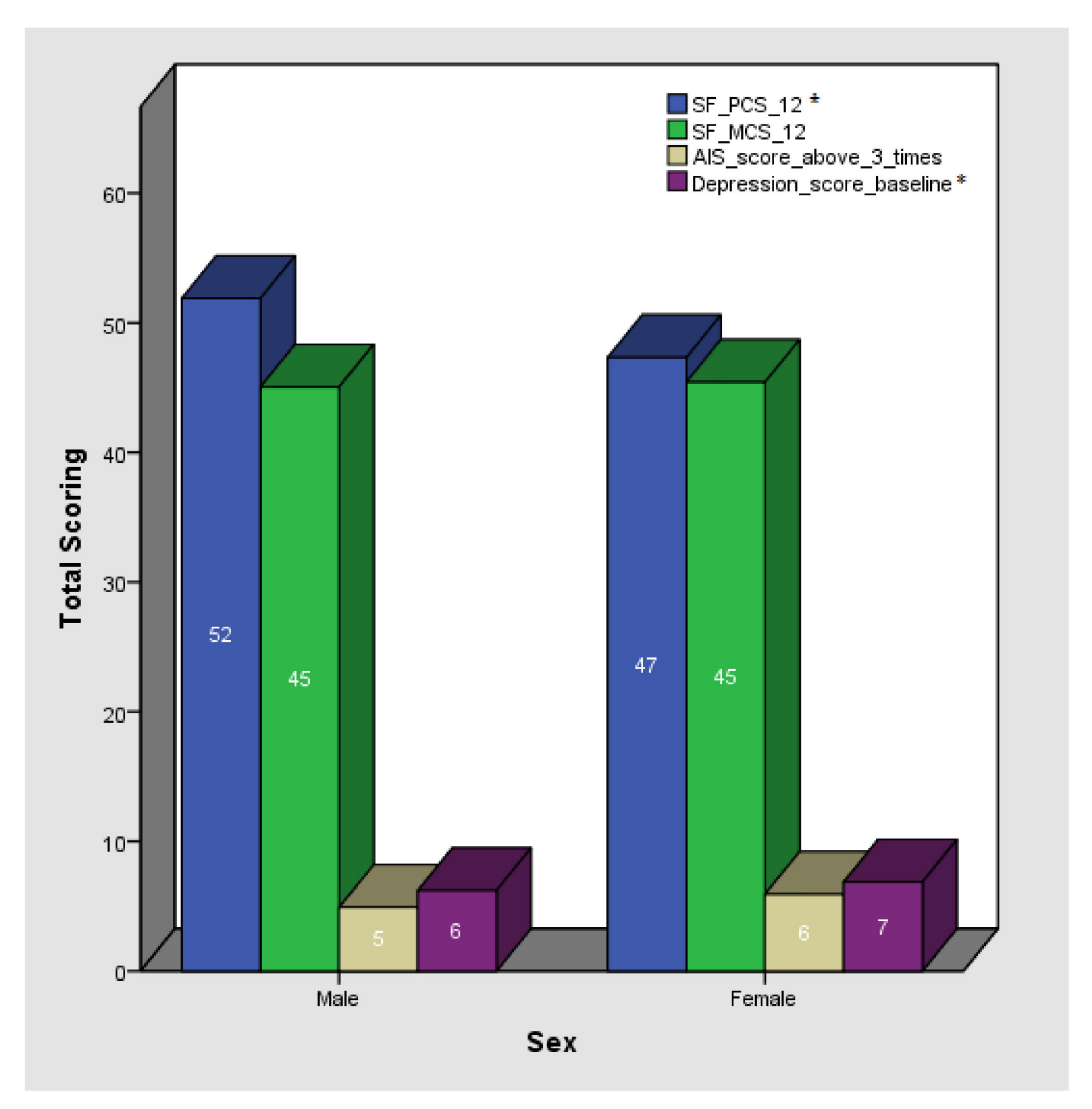
Baseline scoring of the four lifestyle questionnaires, based on sex (* The presented variables were statistically significantly different between the sexes.).

Contrary to the AIS score, the CESD-R-10 scale score on depression characteristics showed that women displayed higher median values (scoring of 6 vs. 5, *p* < 0.001). The majority of the participants did not display depression characteristics, such as feelings of fear and helplessness, with the overall sample presented a mean CESD-R-10 score of 6, with the scale maximum scoring calculated at 18. Moreover, the physical component of the SF-12 score on self-reported quality of life underlined increased levels in men than in women (53, 82 vs. 50, 37, *p* < 0.001), whereas the mental component did not show important dissimilarities. No statistically significant differences were found when the participants were classified into the four different BMI groups (Figure 2 and Figure 3).

As shown in Table 3. we further investigated the potential effect of the aforementioned lifestyle aspects on logBMI and %body fat baseline levels. After adjusting for confounding factors including age, sex, smoking habits, physical activity level and education years, Only the physical component of the SF-12 questionnaire (SF PCS 12 Score) was found to be associated with the characteristics of interest, displaying a negative effect on both logBMI and %body fat values (β *=* −0.003, *p* < 0.001 and β *=* −0.218, respectively).

### 3.3. Dietary Patterns

PCA on the available data of the 202 participants’ FFQs resulted in the identification of five dietary patterns accounting for 40.34% of the sample’s total variance. The KMO and Bartlett’s Test (*p* < 0.001) presented a Kaiser–Meyer–Olkin Measure of 0.726, indicating sufficient data adequacy. All factor loadings for each component were above or approaching a value of 0.3. As shown in Table 4, the 32 food groups formed based on the 69-item questions, included in the analysis reflected the variety of foodstuffs consumed by the sample population, including both widely consumed food categories such as meat and cereals, as well as traditional Greek recipes (i.e., pastitsio, spinach rice, and homemade pies). Alcohol and beer reported servings were included in the analysis, due to the sample’s low median values (2 and 16 mL/d, respectively) and the lack of heavy drinkers.

The dietary patterns provided are summarized in the following (Table 5): (a) The “Mixed” pattern (total variance explained: 14.74%) which reported the consumption of a variety of food groups including both light products and processed products high in fat and sugars (i.e., sweets, mayonnaise, refined cereals, salty snacks, sugary snacks, tray sweets, the Greek pastitsio, potatoes, chicken, seed oil, margarine, butter, light products, and sausage); (b) the “Mediterranean-proxy” (or Med-proxy) pattern (total variance explained: 9.87%), including the consumption of food groups usually found in the Mediterranean diet, such a vegetables, dairy, eggs, fruits, non-refined cereals, large fish, olive oil, dried fruit, and caffeinated drinks, such as coffee and tea; (c) the “Eating out” pattern (total variance explained: 6.26%), consisting of food group combinations frequently consumed outside the household, i.e., seafood, French fries, pies, red meat and alcohol; (d) the “Traditional, vegetarian-alike” pattern (total variance explained: 4.96%), reporting consumption of legumes and traditional Greek recipes (i.e., spinach rice and cooked green peas); and (e) “High in unsaturated fats and fruit juice consumption” pattern (total variance explained: 4.49%), consisting of olives, small fish, nuts and fruit juice, with the first, high in unsaturated fats and fruit juice consumption, groups presenting the greatest factor loadings. 

Nominal associations (*p* < 0.05) are described as follows: consumption of the “Mixed” pattern was correlated with: (a) increased logBMI values, after adjustment for the confounding factors of all models (Model 1:β *=* 0.019, *p* < 0.001, Model 2:β *=* 0.017, *p* < 0.001, Model 3: β *=* 0.015, *p*-value *=* 0.009; (b) increased levels of body fat percentage, in model 1 (β *=* 1.179, *p =* 0.003); (c) increased levels of the logVisceral fat in Model 1 (β *=* 0.032, *p =* 0.001); (d) increased levels of logCreatinine, in Model 3 (β *=* 0.012, *p =* 0.048) (e) decreased values of HDL cholesterol in Model 1 (β *=* −0.020, *p* < 0.006); (f) increased levels of logTriglycerides, in Models 1 (β *=* 0.038, *p =* 0.007) and 3 (β *=* 0.033, *p =* 0.048); (g) increased levels of logSerum protein in Models 1 (β *=* 0.004, *p =* 0.036) and 3 (β *=* 0.005, *p =* 0.029); and (h) increased levels of logSGOT/AST (Model 1: β *=* 0.024, *p =* 0.029, Model 2: β *=* 0.024, *p =* 0.043, Model 3: β *=* 0.028, *p*-value *=* 0.022) and SGPT/ALT (Model 1:β *=* 0.052, *p* < 0.001, Model 2: β *=* 0.049, *p =* 0.002, Model 3: β *=* 0.070, *p* < 0.001). The “Med-proxy” pattern was found related with lower values of logCreatinine, in Model 3 (β *=* −0.013, *p =* 0.029) and lower values of logTotal Bilirubin, in all models (Model 1: β *=* −0.036, *p =* 0.025, Model 2: β *=* −0.043, *p =* 0.010, Model 3: β *=* −0.042, *p =* 0.019). The “Traditional, vegetarian-alike” pattern was associated with: (a) reduced levels of body fat, in Models 1 (β *=* −0.770, *p =* 0.048) and 2 (β *=* −0.476, *p =* 0.032); (b) decreased logVisceral fat values, in Model 2 (β *=* −0.008, *p =* 0.032); and (c) increased levels of logHDL cholesterol in models 2 and 3 (Model 2: β *=* 0.017, *p =* 0.017, Model 3: β *=* 0.016, *p =* 0.039). After evaluation for the adjusted threshold of statistical significance (i.e., a *=* 0.05/5 *=* 0.01), statistically significant associations remained for: (a) the “Mixed” pattern and increased logBMI, body fat and logSGPT/ALT values; (b) the “Mixed” pattern and decreased logHDL cholesterol values; and (c) the “Med-proxy” pattern and decreased levels of logTotal Bilirubin. Further associations are displayed in Appendix A. 

**Figure 4:**
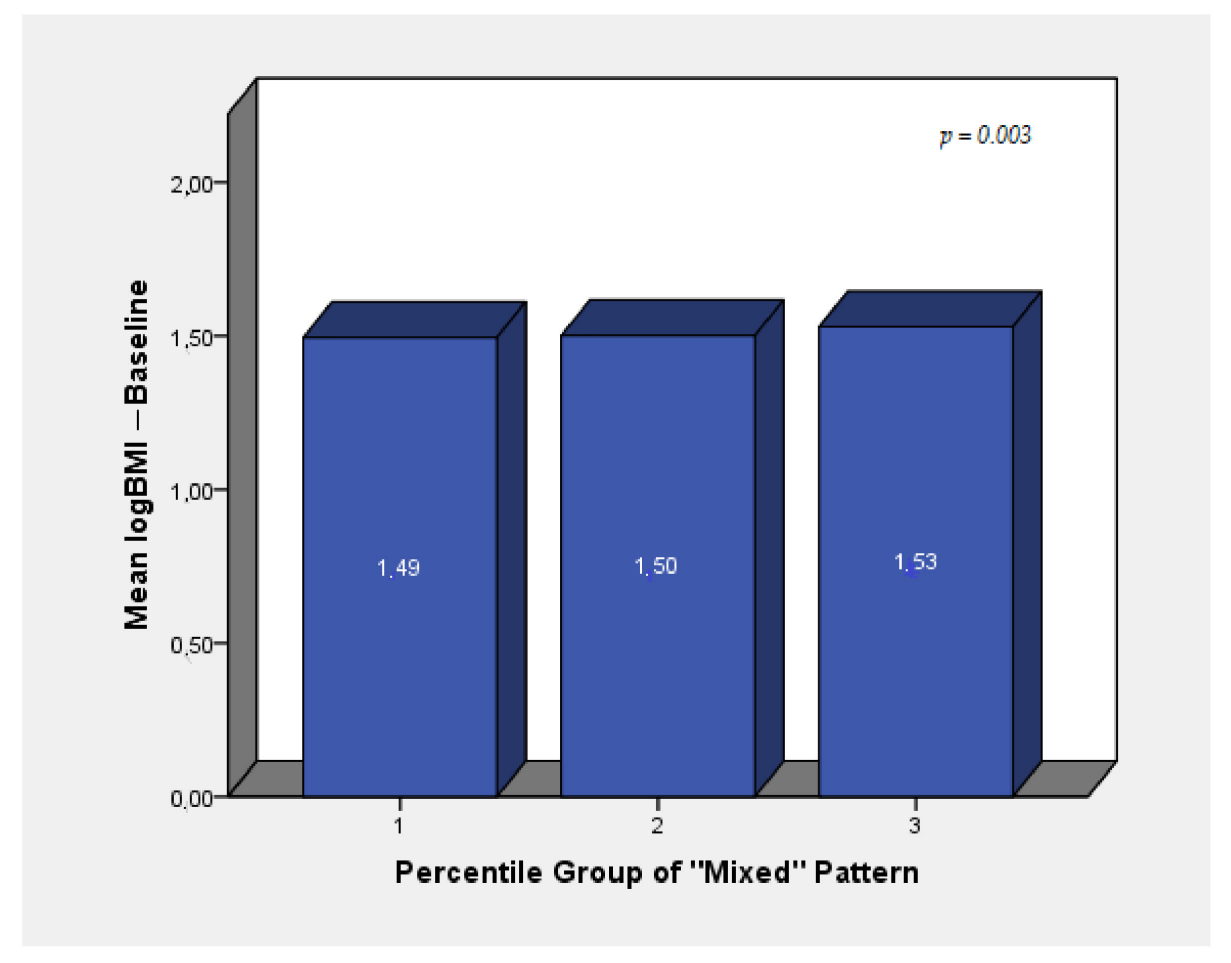
Percentile distribution and associations between the “Mixed” Pattern and logBMI values.

**Figure 5:**
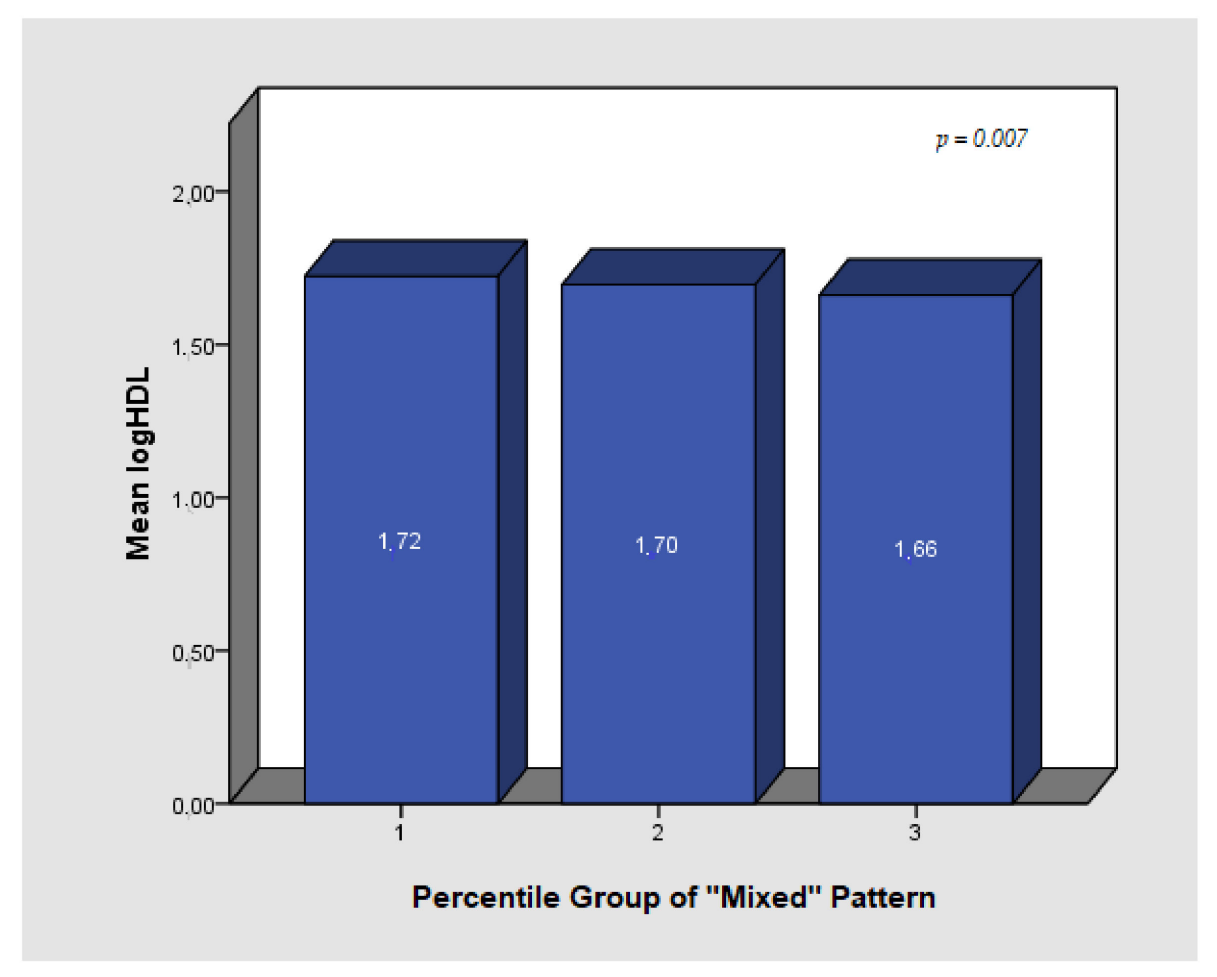
Percentile distribution and associations between the “Mixed” Pattern and logHDL values.

**Figure 6:**
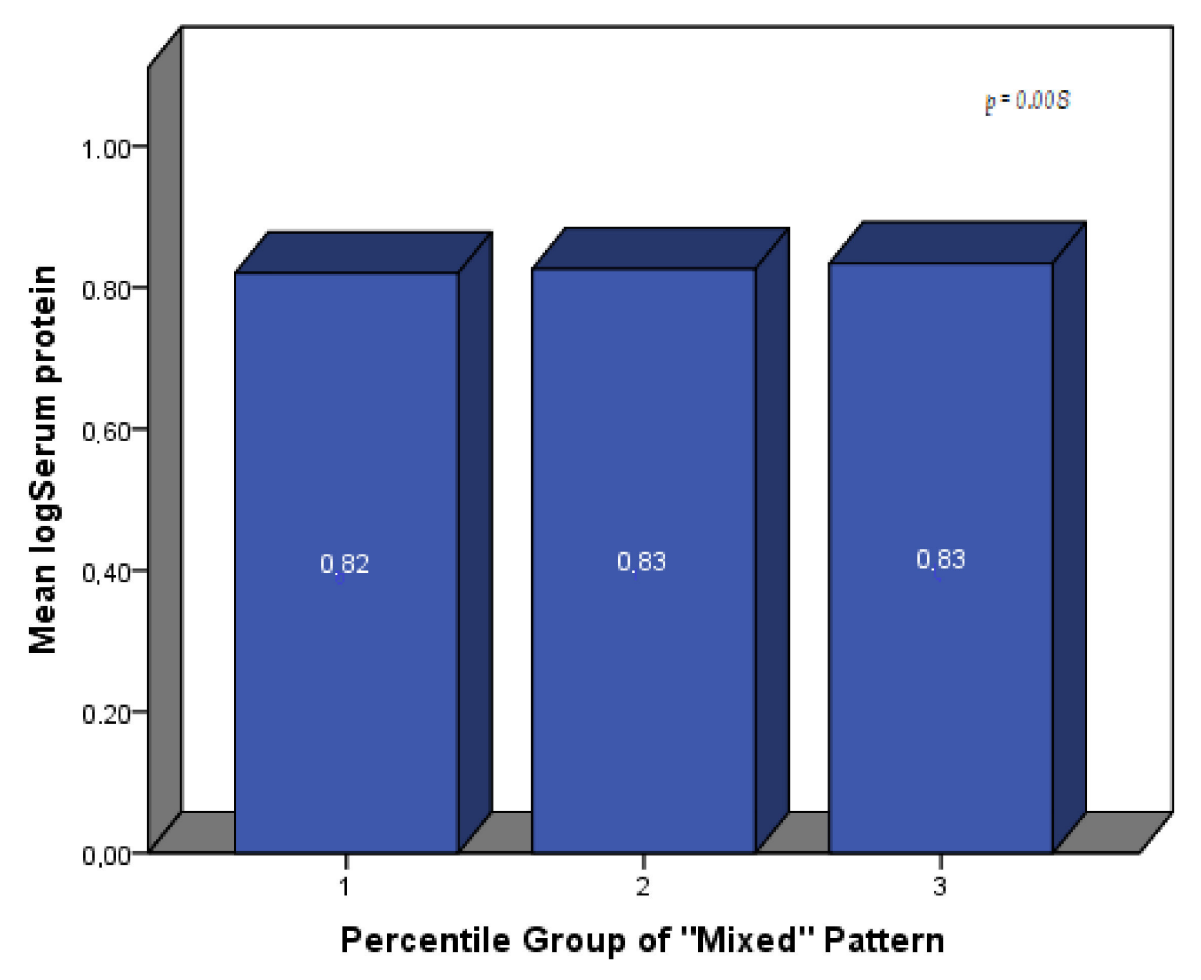
Percentile distribution and associations between the “Mixed” Pattern and logSerum Protein values.

**Figure 7:**
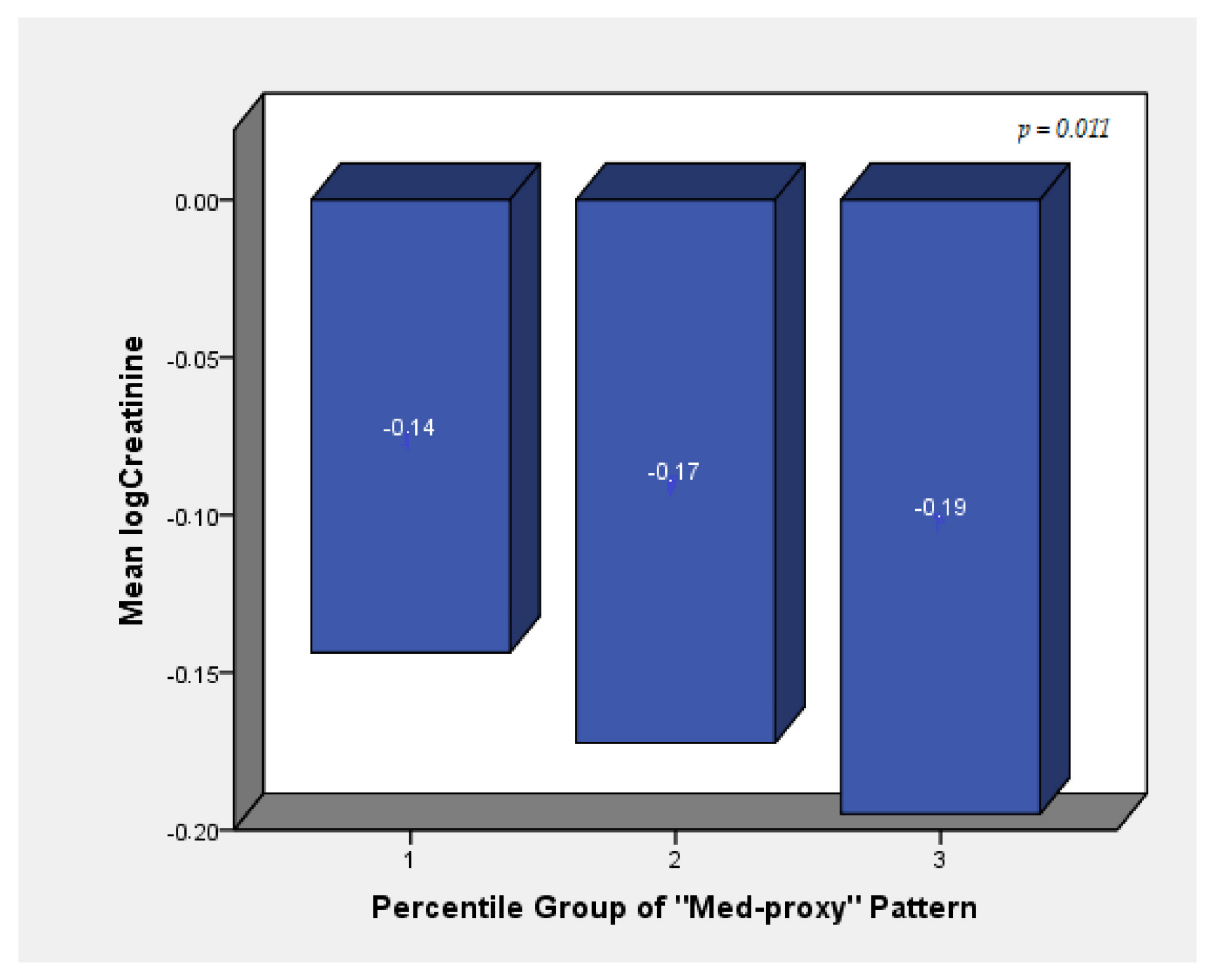
Percentile distribution and associations between the “Med-proxy” Pattern and logCreatinine values.

After extracting the dietary patterns, we further explored the effect of their respective adherence to each anthropometric and biochemical biomarker, by separating them into tertiles. As shown in Figure 4, Figure 5, Figure 6 and Figure 7, increased adherence to the “*Mixed*” pattern was associated with: (a) increased levels of logBMI (*p =* 0.003), (b) decreased levels of logHDL cholesterol (*p =* 0.007), (c) increased levels of logSerum protein (*p =* 0.008). Additionally, categorization in the higher tertile of the “*Med-proxy*” pattern was associated with lower levels of logCreatinine (*p =* 0.011).

### 3.4. Lifestyle Index (LI) Construction

Following extraction of the dietary patterns, we examined the potential relations between different sets of variables, in order to evaluate the construction of a Lifestyle Index. We examined potential correlations between the reported lifestyle questionnaire scores, the extracted dietary patterns, and basic aspects, such as smoking and physical activity habits, with anthropometric indices, such as BMI and body fat percentage. All variables under examination were divided into categories, with higher values indicating favorable effects. Continuous and nominal variables displaying positive correlations were dichotomized based on the sample’s reported median values (attribution of a value of 1 for scores below the sample’s median and a value of 2 for scores above the observed median).

Variables displaying statistically significant (*p* < 0.05), positive correlations with either logBMI of body fat percentage values included: the “Mixed” and “Med-proxy” dietary patterns, the CESD-R-10 depression scale score, and the physical component of the SF-12 scored questionnaire. Subsequently, a Lifestyle Index was created, based on the sum of the aforementioned, dichotomized variables and physical activity categories, as shown in Equation 1. Smoking habits were not included in the Index creation, due to roughly 75% of our sample being reported as non-smokers. Maximum score of the Lifestyle Index was calculated at the value of 11. The Index was calculated for 141 participants and the sample presented a median score of 8 and an IQR of 2.

(1) $$\begin{array}{c} \begin{array}{cc} LifestyleIndex & =Physical\;Activity\;Category+”Mixed”pattern\;dichotomized\;score+”Med\\ & -\;proxy”dichotomized\;score+CESD-R-10\;dichotomized\;score+SF\\ & -\;PCS\;dichotomized\;score \end{array} \end{array}$$

Variables displaying statistically significant (*p* < 0.05), positive correlations with either logBMI of body fat percentage values included: the “Mixed” and “Med-proxy” dietary patterns, the CESD-R-10 depression scale score and the physical component of the SF-12 scored questionnaire. Subsequently, a Lifestyle Index was created, based on the sum of the aforementioned, dichotomized variables and physical activity categories, as shown in Equation 1. Smoking habits were not included in the Index creation, due to roughly 75% of our sample being reported as non-smokers.

As depicted in Table 6, the Lifestyle index presented strong associations, including an inverse correlation with: (a) logBMI (β *=* −0.010. *p*=0.019), (b) logFasting glucose (Model 1: β *=* −0.009, *p =* 0.007. Model 2: β *=* −0.007. *p =* 0.036); and (c) logSGPT (β *=* −0.027, *p =* 0.049). When looking at the sex-stratified analyses. Women displayed negative associations between the Index’s values and body fat percentage (Model 1: β *=* −0.911, *p =* 0.030). logFasting glucose (Model 1: β *=* −0.011, *p =* 0.003. Model 2: β *=* −0.010, *p =* 0.007). logSGOT/AST (Model 1: β *=* −0.017, *p =* 0.026. Model 2: β *=* −0.018, *p =* 0.023) and logSGPT/ALT (Model 1: β *=* −0.039. *p =* 0.003, Model 2: *=* −0.038, *p =* 0.005). Men also showed negative associations with logBMI (β *=* −0.015, *p =* 0.045) and body fat percentage (Model 1: β *=* −1.123, *p =* 0.048).

## 4. Discussion

The present analyses display the design and the baseline population characteristics and dietary habits of the iMPROVE study. Overall, our baseline sample of 202 volunteers displayed satisfactory levels of lifestyle quality, with the majority of participants not reporting depression symptoms or heavily disrupted sleep quality.

Five dietary patterns were identified, including: (a) a “Mixed” pattern; (b) a pattern including food groups similar to those of the Mediterranean diet, entitled “Med-proxy” pattern; (c) the “Eating-out” pattern consisting of food combinations usually found in restaurants or fast-food environments (i.e., pies); (d) the “Traditional, vegetarian-alike” pattern, characterized by plant-based, Greek, traditional recipes; and (e) the “High in unsaturated fats and fruit juice consumption” pattern, including foods groups with high unsaturated fats and magnesium content (i.e., small fish and nuts) and highlighting representative habits of healthy snacking across Greek adults (i.e., olives, nuts and fruit juice). Interestingly, while the “Mixed” pattern included a vast majority of processed foods with added sugars and high fat content (i.e., chocolate, croissants, tray sweets, soft drinks, chips, seed oil, margarine, and butter), it was also characterized by light products and chicken and potatoes’ consumption. This can be potentially attributed to the representative consumption of specific food groups by overweight and obese Greeks, who tend to adhere to short-term, self-imposed attempts to follow a more balanced diet. The latter do not result in successful weight management and/or weight loss efforts, but are exactly characterized by increased consumption of light products and simple food combinations, such as chicken and potatoes. The “Med-proxy” and the “Traditional, vegetarian-alike” patterns are representative of the dietary habits of the Greek population, evidently influenced by the Mediterranean diet and its increased content in fruit, vegetables, and legumes. Apparently, due to its high sugar and fat content, the “Mixed” pattern was associated with higher levels of anthropometric and biochemical characteristics. On the other hand, the plant-based, traditional recipes presented negative associations with body fat and positive relations with increased levels of HDL cholesterol. We further evaluated the within-group tertile categorization of adherence to each pattern, showing that higher tertiles were related to stronger associations for specific patterns, such as the positive relationship between adherence to the “Mixed” pattern and logBMI levels and the negative relationship between the increased adherence to the same pattern and logHDL values.

The concept of obese adults and the effect of dietary intake in the formation of their cardiometabolic profile display great interest, with current literature to be reporting similar findings to the ones outlined in the present study. Interestingly, the majority of studies aiming at identifying dietary patterns in overweight/obese populations, usually provide results for dietary habits adhering to the Western diet (including food groups with increased content of processed foods and/or foods in high fat and sugar content) or to a more balanced dietary pattern including fruit and vegetables, relating to higher and lower values of BMI, respectively [2]. Such patterns may include food combinations each time representative of the region of living, while maintaining a strong influence of the dietary habits and combinations usually found in the Western and/or the Mediterranean diet. A 2021 study by Saghafi-Asi et al. investigating the relationship between dietary patterns and biochemical biomarkers of 151 healthy obese Iranian adults, also underlined a positive association between a “Western” dietary pattern with high fat and sugar content and BMI and body fat levels [22]. Additionally, a different study in Romanian obese adults also underlined the identification of a “high meat/high fat”, a “Western,” and a “Prudent” pattern [23]. Similar findings were reported in a cohort of 410 Polish participants of a case-control study, where adherence to a pattern influenced by the Western one was related with higher levels of fat tissue and waist circumference, in contrast with the adherence to a “Healthy” pattern [24]. In their 2019 longitudinal study, Neri-Sanchez et al. also underlined the positive association between adherence to a “Risky” dietary pattern, including high fat and high sugar content, with the presence of central obesity in Mexican adults [25]. A different pattern consisting of poultry, vegetables, red meat, and red meat products, among others, was also associated with obesity in male, Chinese adults, in a 2021 study of 1739 adults by Wang et al. [26].

Additionally, a different cross-sectional study of our group identified similar associations between dietary patterns and biochemical biomarkers in the adults of the POMAK population. More specifically, the dietary pattern including increased consumption of products high in sugars was related to low levels of HDL cholesterol [27]. Similar trends were also noted when investigating the dietary patterns of adolescent populations, where a dietary pattern with high protein and animal fat content was associated with elevated levels of logBMI and logTriglycerides, in French teenagers [28].

Furthermore, the development of the novel Lifestyle Index using the data deriving from the study sample, allowed for further investigation of the quality of life characteristics on the anthropometric and biochemical indices. Consisting of five variables, including two of the present dietary patterns extracted, the Index displayed negative associations with logBMI and body fat levels, as well as levels of the log-transformed variables of fasting glucose, SGOT, and SGPT. Thus, LI confirmed that higher quality of dietary intake and higher levels of physical activity reduced depression symptoms and improved self-reported conception of health status and may display a protective effect on body composition, as well as a favorable influence on improved glycemic profile.

Overall, development of lifestyle indices as a means of quantifying and evaluating the potential influence of specific lifestyle aspects on body weight is mounting, as analyzed in the beginning of the paper, lifestyle indices can also incorporate dietary information via calculation of diet quality indices. A 2017 systematic review of 34 studies by Asghari et al., sought to investigate the effect of diet quality indices in obesity-related traits, showing that Healthy Eating Index (HEI) displayed an inverse association with obesity. The same review also concluded that different dietary scores, in general, did not efficiently assess diet quality, with most significant findings being presented in populations of the United States [29]. Furthermore, different research groups have investigated the effect of lifestyle characteristics, such as sedentary behavior and screen-time, in adolescent populations [30,31,32]. In adults, current research refers to potential associations between constructed lifestyle indices and specific diseases or disease-related outcomes, namely cardiovascular disease [8], cancer [33], and type 2 diabetes [34]. Lenz et al., showed that creation of a Lifestyle Index for evaluation of life quality in adults at risk for cardiovascular disease can be a useful tool [35]. Furthermore, in a similar effort to evaluate the lifestyle aspects and weight characteristics, Roda et el in 2016, also investigated the potential effect of sleep qualities, screen time, and dietary intake, among others, highlighting a strong positive association between sedentary behavior and overweight [36].

A major advantage of the present study is the use of the online assessment tool, as a means enabling long-distance communication and monitoring, during the time of social distancing, due to the novel coronavirus disease 19 (COVID-19) pandemic. On the other hand, limitations of the present study include: (a) the substantial impact of the COVID-19 pandemic on volunteer recruitment rates. More specifically, conduct of the study’s volunteer recruitment took place exactly in the midst of the COVID-19 pandemic, which resulted in a limited recruitment capacity due to: (a) The social-distancing protocols implemented in recruitment sites, properly adhering to the state guidelines, which resulted in a restricted number of participants visiting the premises; (b) the limited expression of interest for participation in the study, due to fear of in-person meetings and subsequent spread of the COVID-19 disease; (c) the long-distance maintenance of an increased adherence rate to the proposed diets, due to the extended time period between the in-person follow-up meetings; and (d) the proper use of the online assessment tool by older adults who had both limited access and knowledge on the use of state-of-the-art technological devices and online tools.

## 5. Conclusions

Results from the present study suggest that the iMPROVE overweight and obese, adult cohort displays a satisfactory level of lifestyle characteristics and dietary behaviors representative of the overweight and obese Greek population. Assessment of the constructed Lifestyle Index, further solidifies the validity of our findings, highlighting the protective effect of increased lifestyle quality in the formation of elevated body weight. In this spectrum, the findings of the present paper enhance the understanding of overweight/obesity lifestyle determinants in our sample population and lay the ground for the next analysis steps of the iMPROVE study, which focus on assessing the impact of the proposed intervention and the role of candidate genes in various weight-related indices. Assessment of the holistic interplay of gene-lifestyle interactions is of vital importance for the in-depth understanding of nutrigenetic influences in weight loss, as well as in the general context of weight management and/or weight loss maintenance.

## Figures and Tables

**Table 1:** Characteristics of the iMPROVE cohort by sex.

*n*	Smoking		Physical Activity Categories			Diet Group	
	Smokers	Non-Smokers	Low	Moderate	Vigorous	High Carbohydrate/Low-Fat	High Protein
Total	50	151	64	104	31	94	108
Women	38	103	43	76	30	74	68
Men	12	48	21	28	11	20	40

**Table 2:** Anthropometric, clinical, dietary characteristics and characteristics of depression, quality of sleep, and health status in the iMPROVE cohort, by sex.

Variable	Total			Women			Men			*p*-Value
	n	Median	IQR	n	Median	IQR	n	Median	IQR	
Age	202	47	15	142	47.50	14	60	45.78 *	17 *	*p* > 0.05 **
SBP (mmHg)	196	121.00	21	139	117.00	19	57	131.00	21	*p* < 0.001 **
DBP (mmHg)	196	80.84 *	9.86 *	139	78.68 *	9.05 *	57	86.09 *	9.85 *	*p* < 0.001 **
Pulse Rate (Beats per minute)	196	74.99	11.38	139	74.00	16	57	73.00	14	*p* > 0.05 **
Anthropometric Characteristics										
Weight (kg)	202	87.10	26	142	82.80	18	60	100.65	29	*p* < 0.001 **
BMI (kg/m^2^)	202	31.34	6.9	142	31.34	6.9	60	31.33	7.1	*p* > 0.05 **
Body fat (%)	202	37.95 *	7.8 *	142	41.50 *	5.4 *	60	29.55 *	5.8 *	*p* < 0.001 **
Body fat (kg)	202	32.95	13.3	142	34.85	12.8	60	29.05	14.5	*p* < 0.002 **
Fat free mass(kg)	202	52.05	18	142	48.50	7	60	71.20	15	*p* < 0.001 **
Total body water (kg)	202	38.05	13	142	36.35 *	4.03 *	60	52.10	11	*p* < 0.001 **
Visceral fat	202	10.00	6	142	9.74	3.16	60	14.50	5.69	*p* < 0.001 **
Upper body fat (%)	201	36.60	10	141	38.50	8	60	32.10	8	*p* < 0.001 **
Upper body fat (kg)	201	17.60	7	141	17.30	7	60	18.00	7	*p* > 0.05 **
Upper body fat-free mass (kg)	201	28.80	9	141	27.67 *	2.75 *	60	38.00	7	*p* < 0.001 **
Waist circumference (cm)	185	99.00	17	130	96.50	15	55	105.00	17	*p* < 0.001 **
Hip circumference (cm)	185	114.50	14	130	115.75	16	55	114.00	10	*p* > 0.05 **
WHR	185	0.85	0.12	130	0.83	0.09	55	0.92	0.09	*p* < 0.001 **
Biochemical Biomarkers										
Fasting glucose (mg/dL)	193	92.00	10.50	135	92.00	10.00	58	95.00	16.25	*p* < 0.001 **
Urea (mg/dL)	193	28.00	9.00	135	27.00	9.00	58	30.12 *	6.01 *	*p* < 0.001 **
Creatinine (mg/dL)	193	0.68	0.21	135	0.62 *	0.10 *	58	0.85 *	0.12 *	*p* < 0.001 **
Uric acid(mg/dL)	193	4.70	1.45	135	4.30	1.10	58	5.75 *	1.00 *	*p* < 0.001 **
Total cholesterol (mg/dL)	193	177.96 *	33.57 *	135	179.32 *	31.98 *	58	174.79 *	37.13 *	*p* > 0.05 **
HDL cholesterol (mg/dL)	193	49.00	16.50	135	52.00	17.00	58	42.00	14.50	*p* < 0.001 **
LDL cholesterol (mg/dL)	193	105.20	38.70	135	105.00	38.40	58	108.00	42.05	*p* > 0.05 **
Triglycerides (mg/dL)	193	90.00	5.00	135	86.00	51.00	58	105.50	86.50	*p* < 0.001 **
Total bilirubin (mg/dL)	193	0.37	0.23	135	0.35	0.23	58	0.45	0.29	*p* < 0.001 **
Direct bilirubin (mg/dL)	193	0.16	0.08	135	0.15	0.09	58	0.17	0.08	*p* < 0.001 **
Serum protein (g/dL)	193	6.70	0.50	135	6.70	0.45	58	6.60	0.60	*p* > 0.05 **
Serum albumin (g/dL)	193	4.20	0.30	135	4.40	0.50	58	4.20	0.40	*p* < 0.001 **
SGOT/AST (IU/L)	193	16.00	6.00	135	15.00	5.00	58	18.00	5.25	*p* < 0.001 **
SGPT/ALT (IU/L)	192	15.00	11.75	134	13.00	9.00	58	22.00	16.25	*p* < 0.001 **
Lifestyle Characteristics										
AIS Score ***	140	5.00	7.00	97	5.00	7.00	43	4.00	7.00	*p* > 0.05 **
CESD-R-10 Scale	201	6.00	5.00	141	6.00	4.00	60	5.00	5.75	*p* < 0.001 **
SF PCS 12 Score	145	51.98	12	99	50.37	11	46	53.82	8	*p* < 0.001 **
SF MCS 12 Score	145	49.37	15	99	49.44	16	46	46.62 *	8.36	*p* > 0.001 **

* The selected variables follow the normal distribution and are presenting as mean ± standard deviation. ** Statistically significant differences between the sexes were assessed via calculation of the Mann–Whitney test. *** The Athens Insomnia Scale Score was calculated only for participants reporting the referred characteristics ≥3 times/week for the past month. SBP: systolic blood pressure; DBP: diastolic blood pressure; BMI: body mass index; WHR: waist-to-hip ratio; HDL: high-density lipoprotein; LDL: low-density lipoprotein; SGOT/AST: glutamic oxaloacetic transaminase/aspartate transaminase; SGPT/ALT: glutamate pyruvate transaminase blood/alanine transaminase; AIS: Athens Insomnia Scale; CESD-R-10: Center for Epidemiologic Studies Depression Scale Revised—10; SF PCS 12: Short Form (Health Survey) Physical Component Score 12; SF MCS 12: Short Form (Health Survey) Mental Component Score 12.

**Table 3:** Multivariate linear regression analyses on the relation between lifestyle characteristics and BMI and body fat baseline values.

Variable	Model 1 *		
	*β*	*SE*	*p*-Value
**logBMI**			
Athens Insomnia Scale Score	0.001	0.001	0.612
CESD-R-10 Scale	0.002	0.001	0.189
SF PCS 12 Score	−0.003	0.001	<0.001
SF MCS 12 Score	0.001	0.001	0.217
**Body fat (%)**			
Athens Insomnia Scale Score	0.188	0.128	0.143
CESD-R-10 Scale	0.175	0.100	0.083
SF PCS 12 Score	−0.218	0.057	<0.001
SF MCS 12 Score	0.049	0.054	0.371

* Model 1: Adjusting for age, sex, smoking, physical activity level and education years.

**Table 4:** Mean consumption and PCA factor loadings of the 32 FFQ-derived food groups.

			Components				
	Mean Consumption (Median. IQR)	Food Group	1	2	3	4	5
Croissant (g/d)	5.2 (11.56)	Sweets	0.705				
Chocolate (g/d)	12.85 (8.85)						
Tarts (g/d)	10.00 (10.00)						
Ice cream (g/d)	7.66 (24.64)						
Mayonnaise (g/d)	1.11 (2.02) *	Mayonnaise	0.664				
White bread (g/d)	19.28 (17.28)	Refined Cereals	0.643				
Cereals (g/d)	4.28 (4.28)						
White rice (g/d)	10.53 (23.32)						
Barley (g/d)	9.33 (30.00)						
Burger bread (g/d)	3.00 (10.44) *						
Chips (g/d)	4.66 (4.66)	Salty Snacks	0.628				
Crackers (g/d)	1.33 (4.28)						
Honey (g/d)	1.07 (4.66)	Sugary Snacks	0.596				
Soft drinks (mL/d)	28.69 (72.42) *						
Fruit compost (g/d)	7.58 96.66) *						
Tray Sweets (g/d)	10.00 (10.00)	Tray Sweets	0.584				
Pastitsio (g/d)	10.00 (10.00)	Pastitsio	0.493				
Potatoes (boiled. cooked. not fried) (g/d)	11.53 (25.53)	Potatoes (boiled, cooked, not fried)	0.469				
Chicken (g/d)	32.14 (0.00)	Chicken	0.388				
Seed oil (g/d)	3.23 (8.09) *	Seed oil, margarine, butter	0.374				
Margarine (g/d)	1.03 (2.46) *						
Butter (g/d)	0.50 (1.00)						
Light mayonnaise (g/d)	0.71 (1.84) *	Light Products	0.367				
Light cold cuts (g/d)	2.00 (6.42)						
Light soft drinks (g/d)	22.00 (70.71)						
Sausage (g/d)	1.08 (1.45)					−0.342	
Tomatoes (g/d)	64.28 (42.85)	Vegetables		0.640			
Lettuce (g/d)	34.28 (34.28)						
Broccoli (g/d)	21.42 (14.76)						
Spinach (g/d)	6.00 (13.28)						
Full fat milk (mL/d)	43.46 (71.26)	Dairy		0.568			
Low fat milk	51.42 (154.28)						
White cheese (g/d)	6.42 (17.28)						
Eggs (g/d)	10.71 (7.38)	Eggs		0.562			
Oranges (g/d)	36.42 (97.95)	Fruits		0.525			
Apples (g/d)	30.00 (80.66)						
Bananas (g/d)	21.42 (57.61)						
Winter fruit (g/d)	32.14 (86.42)						
Summer fruit	32.14 (64.28)						
Whole bread (g/d)	19.28 (17.28)	Non-refined cereals		0.443			
Brown rice (g/d)	6.72 (14.73) *						
Whole pasta (g/d)	8.54 (9.33)						
Large fish (g/d)	10.00 (22.14)	Large fish		0.432			
Olive oil (g/d)	45.00 (45.00)	Olive oil		0.345			
Dried fruit (g/d)	3.35 (6.88) *	Dried fruit		0.330			
Coffee (mL/d)	240.00 (240.00)	Caffeinated Beverages				−0.504	
Tea (mL/d)	16.00 (51.42)						
Seafood (g/d)	10.00 (10.00)	Seafood			0.685		
French Fries (g/d)	4.83 (15.53)	French Fries			0.648		
Homemade pies (g/d)	10.00 (0.00)	Pies			0.510		
Other pies (g/d)	10.00 (10.00)						
Beef (g/d)	10.00 (22.14)	Red Meat			0.499		
Minced beef	25.71 (17.71)						
Pork (g/d)	10.00 (22.14)						
Lamb (g/d)	5.83 (13.84)						
Alcohol (mL/d)	2.00 (6.42)	Alcohol and Beer			0.398		
Beer (mL/d)	16.00 (51.42)						
Legumes (g/d)	64.28 (44.28)	Legumes				0.698	
Spinach and Rice (g/d)	16.66 (53.57)	Traditional, Greek recipes				0.695	
Green Peas (g/d)	42.85 (29.52)						
Olives	1.00 (3.21)	Olives					0.645
Small fish (g/d)	10.00 (32.14)	Small fish					0.584
Nuts (g/d)	3.33 (28.81)	Nuts					0.343
Fruit Juice (g/d)	16.00 (51.42)	Fruit Juice					0.311
Total Variance Explained (%)			14.74%	9.87%	6.26%	4.96%	4.49%

* The selected variables are presented as mean ± standard deviation (SD).

**Table 5:** Multivariate linear regressions between the extracted dietary patterns and indices of anthropometric and biochemical characteristics.

	Model 1			Model 2			Model 3		
	*β*	*SE*	*p*-Value	*β*	*SE*	*p*-Value	*β*	*SE*	*p*-Value
LogBMI									
Mixed Pattern	0.019	0.005	<0.001	0.017	0.005	0.001	0.015	0.005	0.009
Med-proxy Pattern	−0.002	0.005	0.758	<0.001	0.005	0.937	−0.001	0.006	0.867
Eating-out Pattern	0.004	0.005	0.366	0.004	0.005	0.432	0.001	0.005	0.780
Traditional, vegetarian-alike Pattern	−0.005	0.005	0.272	−0.008	0.005	0.132	−0.008	0.005	0.132
High in unsaturated fats and fruit juice consumption Pattern	−0.005	0.005	0.338	−0.006	0.005	0.269	−0.006	0.005	0.281
Body fat %									
Mixed Pattern	1.179	0.392	0.003	−0.149	0.230	0.516	−0.195	0.259	0.451
Med-proxy Pattern	−0.010	0.423	0.982	0.261	0.234	0.266	0.351	0.255	0.171
Eating-out Pattern	0.316	0.399	0.430	0.005	0.217	0.982	−0.045	0.232	0.848
Traditional, vegetarian-alike Pattern	−0.770	0.387	0.048	−0.476	0.221	0.032	−0.402	0.236	0.090
High in unsaturated fats and fruit juice consumption Pattern	−0.335	0.400	0.404	0.054	0.220	0.808	0.167	0.236	0.480
LogVisceral Fat									
Mixed Pattern	0.032	0.009	0.001	−0.002	0.004	0.633	<0.001	0.004	0.980
Med-proxy Pattern	−0.002	0.010	0.808	0.002	0.004	0.629	0.004	0.004	0.302
Eating-out Pattern	0.011	0.010	0.266	0.002	0.004	0.548	0.002	0.004	0.642
Traditional, vegetarian-alike Pattern	−0.018	0.009	0.058	−0.008	0.004	0.032	−0.007	0.004	0.088
High in unsaturated fats and fruit juice consumption Pattern	−0.007	0.010	0.450	0.002	0.004	0.522	0.006	0.004	0.139
logCreatinine(mg/dL)									
Mixed Pattern	0.008	0.005	0.136	0.008	0.006	0.143	0.012	0.006	0.048
Med-proxy Pattern	−0.008	0.006	0.150	−0.010	0.006	0.066	−0.013	0.006	0.029
Eating-out Pattern	−0.001	0.005	0.897	0.001	0.005	0.848	0.001	0.006	0.875
Traditional, vegetarian-alike Pattern	−0.003	0.005	0.549	−0.008	0.005	0.155	−0.008	0.006	0.149
High in unsaturated fats and fruit juice consumption Pattern	0.002	0.005	0.648	0.001	0.005	0.802	0.001	0.006	0.876
logHDL Cholesterol (mg/dL)									
Mixed Pattern	−0.020	0.007	0.006	−0.013	0.007	0.079	−0.016	0.009	0.057
Med-proxy Pattern	−0.005	0.008	0.528	−0.009	0.008	0.257	−0.010	0.008	0.245
Eating-out Pattern	0.009	0.007	0.229	0.011	0.007	0.135	0.013	0.008	0.082
Traditional, vegetarian-alike Pattern	0.013	0.007	0.054	0.017	0.007	0.017	0.016	0.008	0.039
High in unsaturated fats and fruit juice consumption Pattern	0.005	0.007	0.483	0.006	0.007	0.445	0.003	0.008	0.688
logTriglycerides(mg/dL)									
Mixed Pattern	0.038	0.014	0.007	0.021	0.015	0.155	0.033	0.016	0.048
Med-proxy Pattern	−0.008	0.015	0.579	−0.003	0.015	0.850	−0.002	0.016	0.908
Eating-out Pattern	<0.001	0.014	0.998	−0.003	0.014	0.816	−0.007	0.015	0.622
Traditional, vegetarian-alike Pattern	0.009	0.014	0.509	0.006	0.014	0.662	0.015	0.015	0.316
High in unsaturated fats and fruit juice consumption Pattern	0.002	0.014	0.875	<0.001	0.015	0.976	−0.004	0.016	0.788
logTotal Bilirubin(mg/dL)									
Mixed Pattern	−0.002	0.015	0.913	0.001	0.017	0.971	0.013	0.018	0.472
Med-proxy Pattern	−0.036	0.016	0.025	−0.043	0.017	0.010	−0.042	0.018	0.019
Eating-out Pattern	−0.005	0.015	0.735	−0.001	0.015	0.943	−0.005	0.016	0.772
Traditional, vegetarian-alike Pattern	0.032	0.015	0.031	0.030	0.016	0.059	0.033	0.016	0.047
High in unsaturated fats and fruit juice consumption Pattern	0.018	0.016	0.255	0.015	0.016	0.357	0.016	0.017	0.356
logSerum protein(g/dL)									
Mixed Pattern	0.004	0.002	0.036	0.003	0.002	0.187	0.005	0.002	0.029
Med-proxy Pattern	−0.001	0.002	0.575	−0.001	0.002	0.720	<0.000	0.002	0.829
Eating-out Pattern	−0.001	0.002	0.603	−0.001	0.002	0.599	−0.002	0.002	0.273
Traditional, vegetarian-alike Pattern	0.002	0.002	0.164	0.002	0.002	0.217	0.003	0.002	0.136
High in unsaturated fats and fruit juice consumption Pattern	0.001	0.002	0.525	0.001	0.002	0.530	<0.001	0.002	0.794
LogSGOT/AST(IU/L)									
Mixed Pattern	0.024	0.011	0.029	0.024	0.012	0.043	0.028	0.012	0.022
Med-proxy Pattern	0.003	0.012	0.797	0.004	0.012	0.759	0.001	0.012	0.911
Eating-out Pattern	−0.011	0.011	0.317	−0.009	0.011	0.393	−0.013	0.011	0.216
Traditional, vegetarian-alike Pattern	0.003	0.011	0.811	−0.003	0.011	0.799	−0.004	0.011	0.735
High in unsaturated fats and fruit juice consumption Pattern	0.006	0.011	0.603	0.004	0.012	0.735	0.003	0.011	0.824
logSGPT/ALT (IU/L)									
Mixed Pattern	0.052	0.014	<0.001	0.049	0.016	0.002	0.070	0.016	<0.001
Med-proxy Pattern	−0.009	0.016	0.570	−0.005	0.016	0.768	−0.006	0.017	0.740
Eating-out Pattern	−0.003	0.015	0.816	−0.005	0.015	0.714	−0.009	0.015	0.555
Traditional, vegetarian-alike Pattern	0.002	0.014	0.910	<0.001	0.015	0.996	<0.001	0.015	0.978
High in unsaturated fats and fruit juice consumption Pattern	0.005	0.015	0.724	0.006	0.016	0.701	−0.002	0.016	0.919

Model 1: Adjusting for age and sex. Model 2: Adjusting for age, sex, smoking habits, physical activity level and logBMI (except for logBMI values). Model 3: Adjusting for age, sex, smoking habits, physical activity level, logBMI, education years, family and professional status.

**Table 6:** Multivariate linear regressions between anthropometric and clinical characteristics and the constructed lifestyle index.

				Model 1									Model 2					
	Total *			Women			Men			Total **			Women			Men		
	*β*	*SE*	*p*-Value	*β*	*SE*	*p*-Value	*β*	*SE*	*p*-Value	*β*	*SE*	*p*-Value	*β*	*SE*	*p*-Value	*β*	*SE*	*p*-Value
logBMI Lifestyle Index	−0.010	0.004	0.019	−0.007	0.005	0.183	−0.015	0.007	0.045	−	−	−	−			−		
Body fat (%)																		
Lifestyle Index	−0.867	0.451	0.056	−0.911	0.414	0.030	−1.123	0.551	0.048	−0.307	0.393	0.436	−0.446	0.231	0.056	−0.446	0.339	0.572
logFasting glucose (mg/dL)																		
Lifestyle Index	−0.009	0.003	0.007	−0.011	0.003	0.003	−0.005	0.006	0.429	−0.007	0.003	0.036	−0.010	0.004	0.007	<0.001	0.006	0.953
*logSGOT (IU/L)*																		
Lifestyle Index	−0.006	0.009	0.534	−0.017	0.007	0.026	0.017	0.020	0.397	−0.006	0.010	0.518	−0.018	0.008	0.023	0.015	0.021	0.484
*LogSGPT (IU/L)*																		
Lifestyle Index	−0.027	0.014	0.049	−0.039	0.013	0.003	<0.001	0.023	0.988	0.292	0.277	0.293	−0.038	0.013	0.005	0.003	0.024	0.895

* Model 1: Adjusting for age. ** Model 2: Adjusting for age and logBMI baseline values.

## Data Availability

The data presented in this study are available on request from the corresponding author. The data are not publicly available due to participants’ privacy and ethical restrictions.

## Appendix A

Continuation of Table 5, with analyses of variables that did not display statistically significant changes (to be uploaded as Table A1).

**Table A1:** Multivariate linear regressions between the extracted dietary patterns and indices of anthropometric and biochemical characteristics.

	Model 1			Model 2			Model 3		
	*β*	*SE*	*p*-Value	*β*	*SE*	*p*-Value	*β*	*SE*	*p*-Value
logGlucose (mg/dL)									
Mixed Pattern	0.006	0.004	0.127	0.001	0.004	0.803	0.001	0.004	0.803
Med-proxy Pattern	<0.001	0.004	0.980	0.002	0.004	0.560	0.002	0.004	0.560
Eating-out Pattern	0.002	0.004	0.635	0.001	0.004	0.862	0.001	0.004	0.862
Traditional, vegetarian-alike Pattern	0.003	0.004	0.462	0.001	0.004	0.871	0.001	0.004	0.871
High in unsaturated fats Pattern	−0.003	0.004	0.380	−0.005	0.004	0.222	−0.005	0.004	0.222
logUrea (mg/dL)									
Mixed Pattern	0.004	0.007	0.566	0.002	0.008	0.814	0.001	0.009	0.932
Med-proxy Pattern	0.010	0.008	0.204	0.012	0.008	0.145	0.012	0.009	0.196
Eating-out Pattern	−0.005	0.007	0.498	−0.005	0.007	0.467	−0.008	0.008	0.349
Traditional, vegetarian-alike Pattern	<0.001	0.007	0.989	−0.001	0.008	0.847	−0.004	0.008	0.622
High in unsaturated fats Pattern	−0.002	0.007	0.758	−0.002	0.008	0.791	−0.004	0.008	0.637
logUric Acid(mg/dL)									
Mixed Pattern	0.006	0.006	0.377	−0.006	0.007	0.361	−0.004	0.007	0.628
Med-proxy Pattern	−0.004	0.007	0.585	−0.001	0.007	0.854	<0.001	0.007	0.963
Eating-out Pattern	0.003	0.006	0.603	0.002	0.006	0.749	−0.002	0.006	0.805
Traditional, vegetarian-alike Pattern	−0.002	0.006	0.739	−0.004	0.006	0.506	−0.004	0.007	0.502
High in unsaturated fats Pattern	−0.006	0.006	0.336	−0.006	0.006	0.358	−0.007	0.007	0.331
Total Cholesterol (mg/dL)									
Mixed Pattern	−0.259	2.402	0.914	1.283	2.575	0.619	0.922	2.915	0.752
Med-proxy Pattern	−3.271	2.534	0.198	−2.864	2.622	0.276	−3.629	2.860	0.206
Eating-out Pattern	−0.080	2.394	0.973	−0.274	2.414	0.910	−0.631	2.601	0.809
Traditional, vegetarian-alike Pattern	2.297	2.297	0.323	3.766	2.463	0.128	4.722	2.618	0.073
High in unsaturated fats Pattern	−0.202	2.445	0.934	0.819	2.519	0.746	0.103	2.711	0.970
logLDL Cholesterol (mg/dL)									
Mixed Pattern	−0.007	0.010	0.492	0.005	0.010	0.613	0.002	0.011	0.878
Med-proxy Pattern	−0.008	0.010	0.430	−0.005	0.010	0.642	−0.008	0.011	0.491
Eating-out Pattern	−0.003	0.010	0.751	−0.004	0.009	0.659	−0.006	0.010	0.557
Traditional, vegetarian-alike Pattern	−0.006	0.009	0.539	0.002	0.010	0.816	0.005	0.010	0.625
High in unsaturated fats Pattern	−0.010	0.010	0.321	−0.003	0.010	0.755	−0.003	0.011	0.746
logAlbumin(g/dL)									
Mixed Pattern	0.002	0.004	0.665	0.003	0.004	0.412	0.003	0.002	0.199
Med-proxy Pattern	−0.002	0.004	0.691	−0.002	0.004	0.584	0.002	0.002	0.289
Eating-out Pattern	<0.001	0.004	0.904	0.001	0.004	0.759	<0.001	0.002	0.977
Traditional, vegetarian-alike Pattern	0.003	0.004	0.368	0.003	0.004	0.508	0.003	0.002	0.115
High in unsaturated fats Pattern	−0.002	0.004	0.606	−0.003	0.004	0.441	0.001	0.002	0.733

Model 1: Adjusting for age and sex. Model 2: Adjusting for age, sex, smoking habits, physical activity level, and logBMI (except for logBMI values). Model 3: Adjusting for age, sex, smoking habits, physical activity level, logBMI, education years, family, and professional status.
